# É Justo Contraindicar o Implante de Desfibrilador Cardíaco para Pacientes com Vulnerabilidade Sócioeconômica ou Psicológica?

**DOI:** 10.36660/abc.20240643

**Published:** 2024-11-07

**Authors:** Marcelo de Paula Martins Monteiro, Helena Nogueira Brasil, Carlos Roberto Martins Rodrigues, Eduardo Arrais Rocha

**Affiliations:** 1 Universidade Federal do Ceará Faculdade de Medicina Fortaleza CE Brasil Faculdade de Medicina da Universidade Federal do Ceará – Cardiologia, Fortaleza, CE – Brasil; 2 Universidade Federal do Ceará Hospital Universitário Walter Cantídio Fortaleza CE Brasil Universidade Federal do Ceará - Hospital Universitário Walter Cantídio, Fortaleza, CE – Brasil; 3 Universidade Federal do Ceará Fortaleza CE Brasil Universidade Federal do Ceará, Fortaleza, CE – Brasil; 4 Centro de Arritmia do Ceará Fortaleza CE Brasil Centro de Arritmia do Ceará, Fortaleza, CE – Brasil

**Keywords:** Desfibrilador Cardíaco, Insuficiência Cardíaca, Vulnerabilidade em Saúde

Os autores Cavalcanti et al.^1^ publicaram um artigo demonstrando que os pacientes submetidos à implantes de desfibriladores cardíacos internos (CDI) por prevenção secundária, com fração de ejeção (FE)<50% e que tinham elevada vulnerabilidade sócioeconômica ou psicológica apresentaram alta mortalidade em 1 ano. Os autores analisaram 4 variáveis de marcadores econômicos e psicossociais (MEPS): 1-vulnerabilidade socioeconômica; 2- capacidade de autocuidado; 3- adesão farmacológica; 4- transtornos do humor. Não ocorreram óbitos em 1 ano no grupo sem a presença dessas variáveis. O estudo englobou uma rede multidisciplinar de análise pré-implante, denominada *device-team.*

A importância do estudo está em identificar alguns novos marcadores de pior prognóstico após esse procedimento, em particular em determinadas áreas populacionais. Os fatores que são passíveis de modificação devem ser rigorosamente acompanhados e modificados, possibilitando melhora evolutiva e garantindo o benefício de aumento da sobrevida que os CDI proporcionam.

Outro fato relevante do trabalho é implementar na tomada de decisões uma equipe multidisciplinar chamada de *device-team.* Não ficou claro no estudo se a equipe teve poder de intervir sobre a decisão ou a negativa ao procedimento, ou apenas foram responsáveis pela coleta e análise dos dados para que, em um segundo momento, fossem passíveis de atuação e modificação. As medidas implementadas após as constatações das variáveis estudadas também não ficaram claras. Apesar de ser um estudo observacional, como foi prospectivo, é provável que diante dos achados, medidas modificadoras tenham sido realizadas.

A utilização de uma equipe multidisciplinar para avaliar esses pacientes antes do implante é uma prática inovadora e essencial para uma decisão mais holística, sendo uma recomendação relevante para contextos de saúde pública onde os recursos são limitados. A inclusão de profissionais de diversas áreas proporciona uma visão integral do paciente, o que é crucial para decisões de alto custo e impacto, como o implante de CDI.

A negativa do benefício ao procedimento para esses casos levanta, entretanto, questões éticas e sociais profundas, principalmente em um país como o Brasil com grande desigualdade socioeconômica. Em bioética, justiça é dar a cada um o que é seu por direito, de forma adequada à pessoa. Uma injustiça ocorre ao negar a uma pessoa algo a que tem direito, ou distribuí-lo de maneira desigual.^2^

Algumas questões merecem reflexões sobre o estudo, como a elevada taxa de mortalidade geral (25,8%). Cardinalli-Neto et al.^3^ demonstraram elevada mortalidade em pacientes pós implantes de CDI em um grupo com muito elevada taxa de choques, provavelmente por programação dos CDI não ideal para esses casos. As programações dos CDI devem seguir normas técnicas rigorosas visando evitar terapias desnecessárias.^4^

Diversos outros estudos diferem dessas elevadas taxas de mortalidade apresentadas em 1 ano. Pereira et al. demonstraram taxa de 6,2% de mortalidade anual em um grupo com 87% de implantes por prevenção secundária, também de regiões em desenvolvimento do Brasil.^5^ Esses dados foram confirmados em outras análises mais detalhadas quando comparadas diferentes etiologias como cardiomiopatias chagásicas (CCC), dilatadas (CNI)^6^ e isquêmicas (CI).^7^ A população com CCC e CDI tem mais terapias, principalmente por terapia antitaquicardia, maior gravidade, porém, sem maior mortalidade súbita. Na CCC com CDI, a baixa escolaridade e a FE < 30 % são fatores preditores de pior evolução.^8^

Quanto aos transtornos do humor, há dados de literatura que demonstram forte relação entre transtorno bipolar e mortalidade súbita com aumento de incidência muito expressivo de arritmias fatais em jovens portadores de bipolaridade, com provável ligação entre a atividade cerebral assimétrica e o tônus autonômico eferente cardíaco.^9^

Outro fator de mau prognóstico verificado no estudo foi a situação econômica dos pacientes. Liu et al.^10^ estudaram pacientes por 12 anos e observaram grande disparidade de mortalidade entre o grupo no quartil inferior de renda anual e o quartil superior. Em média, se verificou a perda de aproximadamente 10 anos de expectativa de vida devido apenas à diferença de renda. Este mesmo estudo revelou que mudanças de estilo de vida foram capazes de mitigar as perdas relacionadas à falta de recursos financeiros.

As causas dos óbitos não foram demonstradas, o que dificulta uma conclusão e extrapolação dos dados, assim como o número de terapias do CDI, as medicações usadas durante o seguimento, as taxas de perda de acompanhamento ou as intercorrências ocorridas, que apesar de não serem objetivo do estudo, dificultam muito a compreensão do que ocorreu com essa população. Sabe-se que o número elevado de choques pode determinar piora na função ventricular e na evolução, conforme demonstrado nos subgrupos do estudo Madit II.^11^

Martinelli et al.^12^ demonstraram taxas de mortalidade anual de 7,1%, assim como de 9% na metanálise de Rassi et al.^13^ em pacientes com CCC. Sabe-se que a CCC apresenta maior gravidade e complexidade, entretanto isso não representou a causa da elevada mortalidade no presente trabalho.

A predição de risco de mortalidade na insuficiência cardíaca crônica (ICC) é complexa. A maioria dos estudos avaliou a predição de hospitalização por ICC e não de mortalidade. A FE passou a ser o parâmetro mais próximo do "Santo Graal", mas quando se dicotomiza entre etiologias isquêmicas e não isquêmicas (CNI), ela perde poder estatístico na CNI.^14^

A assistência médica por si é um ato que não pode ser dissociado de uma ampla rede de amparo socioeconômico e psíquico, uma informação que o presente estudo demonstra, sendo talvez o ponto mais alto do trabalho. A assistência integral é primordial principalmente nesses grupos de alto risco e que foram submetidos a procedimentos de alta complexidade.

Dessa forma o estudo se mostrou importante pelo debate de um tema complexo, entretanto se precisam mais detalhes do que ocorreu com essa população para efetivas conclusões, devendo ter cuidado para que não sejam privados de procedimentos de alta complexidade doentes com maior vulnerabilidade socioeconômica e psicologia. O ideal é que consigamos efetivamente uma ampliação na rede de cuidados e assistência que beneficie esses grupos mais vulneráveis da mesma forma como é demonstrado na literatura nacional e mundial.

A consideração ética de contraindicar tratamentos com base em vulnerabilidades socioeconômicas ou psicológicas também deve ser rigorosamente debatida para assegurar que a prática médica não exacerbe as desigualdades existentes. Acreditamos que mais estudos são necessários, que incluam amostras mais diversificadas e avaliem intervenções que possam reduzir o impacto negativo dos MEPS na sobrevida dos pacientes, promovendo uma medicina mais equitativa e não de exclusão.

## References

[1] Cavalcante WC, Viana TT, Figueiredo CS, Martins F, Passos LCS (2024). Potentially Inappropriate Cardioverter Defibrillator Implants in Secondary Prevention of Death. Arq Bras Cardiol.

[2] Aparisi JCS (2010). Los Principios de la Bioética y el Surgimiento de una Bioética Intercultural. Veritas.

[3] Cardinalli A, Bestetti RB, Cordeiro JA, Rodrigues VC (2007). Predictors of All-Cause Mortality for Patients with Chronic Chagas’ Heart Disease Receiving Implantable Cardioverter Defibrillator Therapy. J Cardiovasc Electrophysiol.

[4] Stiles MK, Fauchier L, Morillo CA, Wilkoff BL (2020). 2019 HRS/EHRA/APHRS/LAHRS Focused Update to 2015 Expert Consensus Statement on Optimal Implantable Cardioverter-defibrillator Programming and Testing. Heart Rhythm.

[5] Pereira FTM, Rocha EA, Gondim DSP, Almeida RLF, Pires RDJ (2024). Predictors of Appropriate Therapies and Death in Patients with Implantable Cardioverter-Defibrillator and Chronic Chagas Heart Disease. Arq Bras Cardiol.

[6] Gondim FTP (2024). Estudo Comparativo dos Portadores de Cardiopatia Chagásica Crônica versus Cardiopatia Não-isquêmica com Desfibrilador Cardíaco Implantável [dissertation].

[7] Pereira FT, Rocha EA, Monteiro MP, Lima NA, Rodrigues CR, Pires RJ (2016). Clinical Course after Cardioverter-defibrillator Implantation: Chagasic versus Ischemic Patients. Arq Bras Cardiol.

[8] Pereira FT, Rocha EA, Monteiro MP, Rocha AC, Daher EF, Rodrigues CR (2014). Long-term Follow-up of Patients with Chronic Chagas Disease and Implantable Cardioverter-defibrillator. Pacing Clin Electrophysiol.

[9] Levenberg K, Critchley HD, Richard DL (2023). Understanding the Mechanisms of Sudden Cardiac Death in Bipolar Disorder: Functional Asymmetry in Brain-heart Interactions as a Potential Culprit. Medical Hypotheses.

[10] Liu L, Wen W, Shrubsole MJ, Lipworth LE, Mumma MT, Ackerly BA (2024). Impacts of Poverty and Lifestyles on Mortality: A Cohort Study in Predominantly Low-income Americans. Am J Prev Med.

[11] Daubert JP, Zareba W, Cannom DS, McNitt S, Rosero SZ, Wang P (2008). Inappropriate Implantable Cardioverter-defibrillator Shocks in MADIT II: Frequency, Mechanisms, Predictors, and Survival Impact. J Am Coll Cardiol.

[12] Martinelli M, Siqueira SF, Sternick EB, Rassi A, Costa R, Ramires JA (2012). Long-term Follow-up of Implantable Cardioverter-defibrillator for Secondary Prevention in Chagas’ Heart Disease. Am J Cardiol.

[13] Rassi FM, Minohara L, Rassi A, Correia LCL, Marin JA, Rassi A (2019). Systematic Review and Meta-analysis of Clinical Outcome after Implantable Cardioverter-Defibrillator Therapy in Patients with Chagas Heart Disease. JACC Clin Electrophysiol.

[14] Rocha EA, Cunha BL, Brasil HN, Pereira FTM, Pires RDJ (2023). Mortality Risk Stratification in Heart Failure. The Search for the Holy Grail Continues! Autonomic Nervous System Analysis is Back!. Arq Bras Cardiol.

