# Supplementary material for: É Justo Contraindicar o Implante de Desfibrilador Cardíaco para Pacientes com Vulnerabilidade Sócioeconômica ou Psicológica?
Source: Arq Bras Cardiol. 2024 Nov 7;121(10):e20240643. [Article in Portuguese] doi: 10.36660/abc.20240643 (PMC11634218; doi:10.36660/abc.20240643)
Supplement: Translation

# Translation: Is it Fair to Contraindicate Cardiac Defibrillator Implantation for Patients with Socioeconomic or Psychological Vulnerability?

**DOI:** 10.36660/abc.20240643i

**Authors:** Marcelo de Paula Martins Monteiro, Helena Nogueira Brasil, Carlos Roberto Martins Rodrigues, Eduardo Arrais Rocha

**Affiliations:** 1 Fortaleza CE Brazil Faculdade de Medicina da Universidade Federal do Ceará – Cardiologia, Fortaleza, CE – Brazil; 2 Fortaleza CE Brazil Universidade Federal do Ceará - Hospital Universitário Walter Cantídio, Fortaleza, CE – Brazil; 3 Fortaleza CE Brazil Universidade Federal do Ceará, Fortaleza, CE – Brazil; 4 Fortaleza CE Brazil Centro de Arritmia do Ceará, Fortaleza, CE – Brazil

**Keywords:** Cardiac Defibrillator, Heart Failure, Health Vulnerability

The authors Cavalcanti et al.^1^ published an article demonstrating that patients undergoing implantation of internal cardiac defibrillators (ICD) for secondary prevention, with ejection fraction (EF) <50%, and who had high socioeconomic or psychological vulnerability presented high mortality in 1 year. The authors analyzed four variables of economic and psychosocial markers (EPSM): 1-socioeconomic vulnerability; 2-self-care capacity; 3-pharmacological adherence; 4- mood disorders. There were no deaths in 1 year in the group without the presence of these variables. The study encompassed a multidisciplinary network of pre-implant analysis called Device-team.

The importance of the study lies in identifying some new markers of worse prognosis after this procedure, particularly in certain population areas. Factors that are subject to modification must be rigorously monitored and modified, enabling evolutionary improvement and ensuring the benefit of increased survival that ICDs provide.

Another relevant fact of the study is the implementation of a multidisciplinary team (MDT) called a Device-team in decision-making. It was not clear in the study whether the team had the power to intervene in the decision or the refusal to proceed or whether they were only responsible for collecting and analyzing the data so that, at a later stage, they could be acted upon and modified. The measures implemented after the findings of the variables studied were also not clear. Despite being an observational study, as it was prospective, it is likely that, given the findings, modifying measures were implemented.

The use of a MDT to evaluate these patients before implantation is an innovative and essential practice for a more holistic decision and is a relevant recommendation for public health contexts where resources are limited. The inclusion of professionals from different areas provides a comprehensive view of the patient, which is crucial for high-cost and high-impact decisions, such as ICD implantation.

The denial of benefits for the procedure in these cases, however, raises profound ethical and social questions, especially in a country like Brazil with great socioeconomic inequality. In bioethics, justice is giving each person what is rightfully theirs in a way that is appropriate to the person. An injustice occurs when a person is denied something to which they are entitled or when it is distributed unequally.^2^

Some issues deserve reflection on the study, such as the high overall mortality rate (25.8%). Cardinali et al.^3^ demonstrated high mortality in patients after ICD implants in a group with a very high shock rate, probably due to ICD programming that was not ideal for these cases. ICD programming must follow strict technical standards to avoid unnecessary therapies.^4^

Several other studies differ from these high mortality rates reported in 1 year. Pereira et al. demonstrated an annual mortality rate of 6.2% in a group with 87% of implants for secondary prevention, also from developing regions of Brazil.^5^ These data were confirmed in other more detailed analyses when comparing different etiologies such as Chagas cardiomyopathy (CCC), dilated cardiomyopathy (DCM),^6^ and ischemic cardiomyopathy (IC).^7^ The population with CCC and ICD has more therapies, mainly by antitachycardia pacing, with greater severity but without greater sudden mortality. In CCC with ICD, low education and EF <30% are predictors of worse outcomes.^8^

Regarding mood disorders, there are data in the literature that demonstrate a strong relationship between bipolar disorder and sudden mortality, with a very significant increase in the incidence of fatal arrhythmias in young people with bipolar disorder, with a probable link between asymmetric brain activity and cardiac efferent autonomic tone.^9^

Another factor of poor prognosis found in the study was the economic situation of the patients. Liu et al.^10^ studied patients for 12 years and observed a large disparity in mortality between the group in the lowest quartile of annual income and the highest quartile. On average, a loss of approximately 10 years of life expectancy was found due to the difference in income alone. This same study revealed that lifestyle changes were able to mitigate losses related to lack of financial resources.

The causes of death were not demonstrated, which makes it difficult to draw conclusions and extrapolate the data, as well as the number of ICD therapies, medications used during follow-up, loss-to-follow-up rates or complications that occurred, which, although not the objective of the study, make it very difficult to understand what happened to this population. It is known that a high number of shocks can determine the worsening of ventricular function and progression, as demonstrated in the subgroups of the Madit II study.^11^

Martino et al.^12^ demonstrated annual mortality rates of 7.1%, as well as 9% in the meta-analysis by Rassi et al.^13^ in patients with CCC. It is known that CCC presents greater severity and complexity; however this did not represent the cause of the high mortality in the present study.

Predicting mortality risk in chronic heart failure (CHF) is complex. Most studies have evaluated the prediction of hospitalization for CHF rather than mortality. EF has become the closest parameter to the "Holy Grail", but when dichotomized between ischemic and non-ischemic etiologies (NIE), it loses statistical power in NIE.^14^

Medical care itself is an act that cannot be dissociated from a broad network of socioeconomic and psychological support, information that this study demonstrates and is perhaps the highlight of the work. Comprehensive care is essential, especially in these high-risk groups that have undergone overly complex procedures.

In this way, the study proved to be important for the debate on a complex topic; however, more details of what happened to this population are needed to draw effective conclusions, and care must be taken so that patients with greater socioeconomic and psychological vulnerability are not deprived of highly complex procedures. Ideally, we should effectively expand the care and assistance network that benefits these more vulnerable groups in the same way as demonstrated in national and international literature.

The ethical consideration of contraindicating treatments based on socioeconomic or psychological vulnerabilities should also be rigorously debated to ensure that medical practice does not exacerbate existing inequalities. We believe that more studies are needed, including more diverse samples and evaluating interventions that can reduce the negative impact of EPSM on patient survival, promoting a more equitable and non-exclusionary medicine.
